# Urinary Bladder Hemangioma Successfully Treated by Angioembolization with Long-Term Follow-Up: Case Report and Literature Review

**DOI:** 10.3390/diagnostics13050875

**Published:** 2023-02-25

**Authors:** Omar Safar, Abdulhadi Al-Qahtani, Saad Al-Qahtani

**Affiliations:** 1 Urology Department, Armed Forces Hospitals Southern Region, Khamis Mushayte P.O. Box 101, Saudi Arabia; 2 Radiology Department, Armed Forces Hospitals Southern Region, Khamis Mushayte P.O. Box 101, Saudi Arabia

**Keywords:** angioembolization, A-V malformation, urinary bladder hemangioma, long-term follow-up

## Abstract

Hemangiomas are benign blood vessel and capillary tumor growths which are widespread in many organs but extremely rare in the bladder, making up just 0.6% of all bladder tumors. To the best of our knowledge, few cases of bladder hemangioma are associated with pregnancy in the literature, and no bladder hemangiomas have been discovered incidentally after abortion. The use of angioembolization is well established; however, postoperative follow-up is crucial to identify tumor recurrence or residual disease. Case presentation: In 2013, a 38-year-old female was referred to a urology clinic with an incidental finding after an abortion of a large bladder mass identified incidentally using ultrasound (US). The patient was recommended for CT, which reported a polypoidal hypervascular lesion, as previously described arising from the urinary bladder wall. Diagnostic cystoscopy showed a large, bluish-red, pulsatile, vascularized submucosal mass with large dilated submucosal vessels, a wide-based stalk, and no active bleeding in the posterior wall of the urinary bladder, measuring about 2 × 3 cm, with negative urine cytology. Due to the vascular nature of the lesion and no active bleeding, the decision was made not to biopsy. The patient underwent angioembolization and scheduled for US every six months with regular diagnostic cystoscopy. In 2018, at 5 years of follow-up, the patient developed recurrence after a successful pregnancy. The angiography revealed recanalization of the previously embolized left superior vesical arteries from the anterior division of the left internal iliac artery, resulting in arteriovenous malformation (AVM). The second angioembolization was performed, with the total exclusion of AVM without residual. By the end of 2022, the patient had remained asymptomatic and without recurrence. Conclusion: Angioembolization is a safe treatment technique, minimally invasive, and has less effect on the quality of life, especially in young patients. Long-term follow-up is essential for detecting tumor recurrence or residual disease.

## 1. Introduction

Hemangiomas are benign blood vessel and capillary tumor growths widespread in many organs but extremely rare in the urinary bladder, where they make up just 0.6% of all bladder tumors [1,2]. Fewer than 100 cases of histologically confirmed hemangiomas of the urinary bladder have been recorded [1]. Congenital in nature, they most likely develop from embryonic angioblastic stem cells [3]. During angioembolization (AE), interventional radiologists approach the peripheral arteries using imaging guidance to cut off the blood flow to specific areas selectively [4]. The role of AE has been recognized in the management of hemorrhagic situations in urology [4]. Herein we presented a rare case of large urinary bladder hemangioma treated successfully using angioembolization with long-term follow-up.

## 2. Case Report

**Figure 1:**
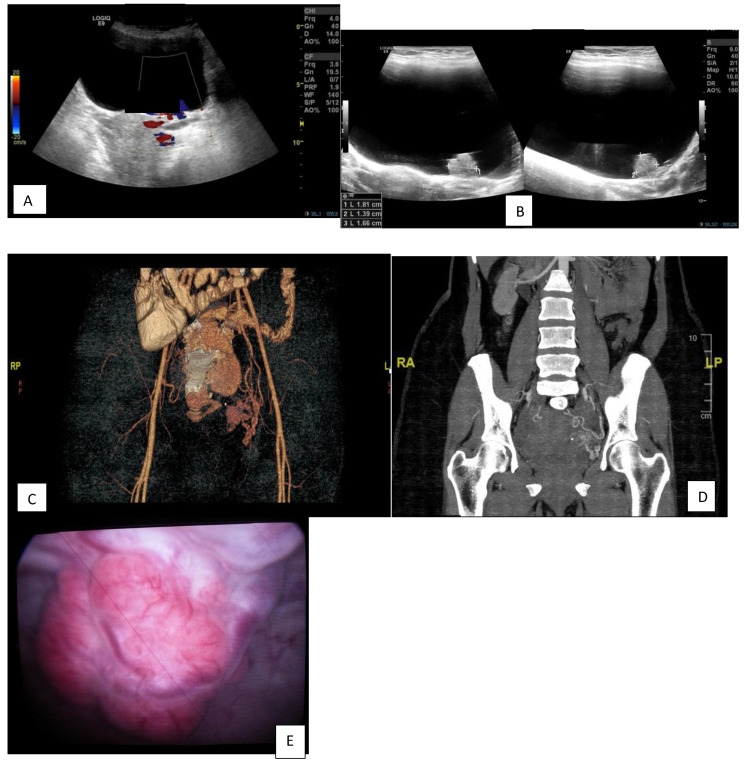
Lesion arising from the urinary bladder wall and diagnostic cystoscopy. (**A**): An ultrasound shows a Pepsi sign on the color Doppler. (**B**): an ultrasound grey scale shows a lesion from the urinary bladder wall. (**C**,**D**): Ct with Iv contrast and processed image of the CT shows a hyper-vascular lesion in the urinary bladder wall. (**E**): a large, pulsatile, submucosal mass and a wide-based stalk in Diagnostic Cystoscopy.

A 38-year-old, medically free female, was referred to a urology clinic in 2013 with an incidental finding of a large bladder mass by ultrasound after an abortion (Figure 1A). The patient had a history of lower urinary tract symptoms with no hematuria. However, her mother had previously been diagnosed with a bladder mass with non-documented pathology and had undergone radical cystectomy.

Computed tomography (CT) with intravenous contrast medium in 2013: A 17.1 × 24.2 mm, well-defined, homogenous hypo- to isodense polypoidal soft tissue lesion was revealed, arising from the posterior wall of the urinary bladder to the left of the midline and bulging inside the urinary bladder. After IV contrast injection, the lesion showed significant contrast enhancement with mild heterogeneity at its base. The rest of the urinary bladder wall was unremarkable.

Impression: A polypoidal hypervascular lesion, as previously described, arising from the urinary bladder wall (and thus unlikely to be arising from the anterior wall of the uterus, see Figure 1B).

The patient then underwent diagnostic cystoscopy, which showed a large, bluish-red, pulsatile, vascularized submucosal mass with large dilated submucosal vessels, a wide-based stalk, and no active bleeding in the posterior wall of the urinary bladder, measuring about 2 × 3 cm, with urine cytology negative (Figure 1C). Due to the vascular nature of the lesion and no active bleeding, the decision was made not to biopsy.

In the same admission, the patient underwent her first angioembolization of the left superior vesical arterial branch approaching from the anterior division of the left internal iliac artery, and was then scheduled for an ultrasound every six months with regular diagnostic cystoscopy. Table 1 summarizes the case management at the time of the presentation.

**Figure 2:**
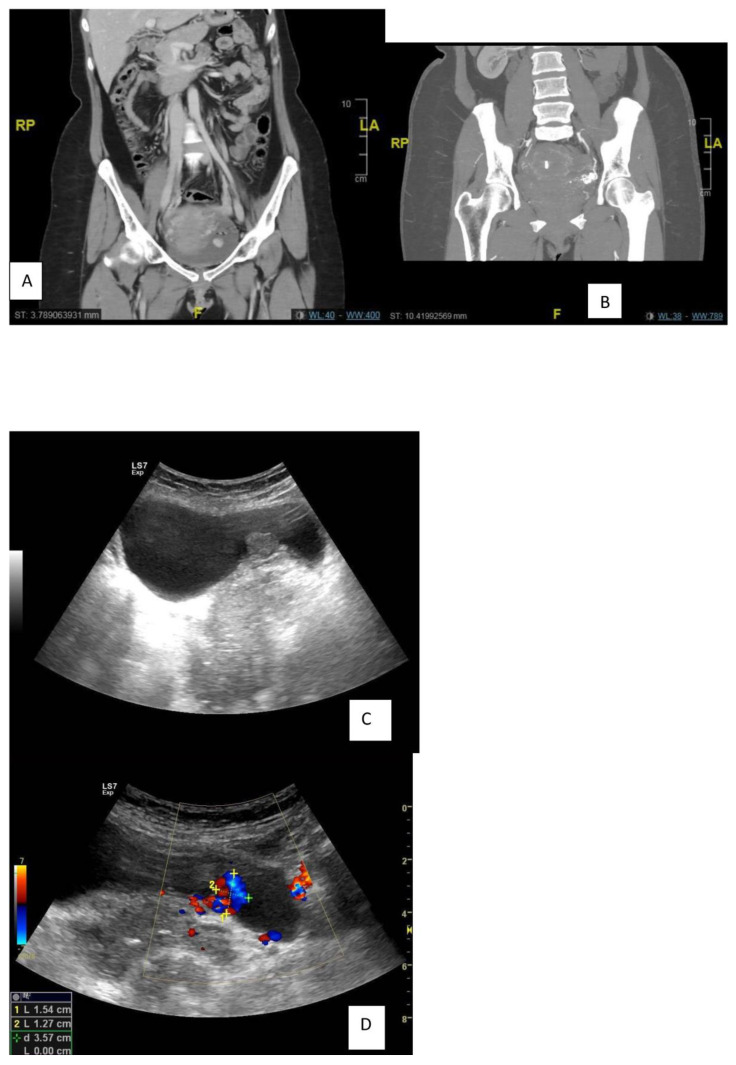
Recurrence after 5 years of follow-up. (**A**,**B**): CT showed a hypervascular lesion in the same site of the previously embolized AVM in the posterior urinary bladder wall (**C**,**D**): Follow up ultrasound Grey scale and color doppler.

After 5 years of follow-up, the patient developed recurrence after a successful pregnancy in 2018: Ultrasonography (US) showed a distended UB with a polypoidal soft tissue mass measuring 16 × 16 mm with internal vascularity, nidus in the afferent and efferent vessels, and turbulence in the flow. CT showed a polypoidal hypervascular lesion in the same site of the previously embolized AVM in the posterior urinary bladder wall [Figure 2A,B].

**Figure 3:**
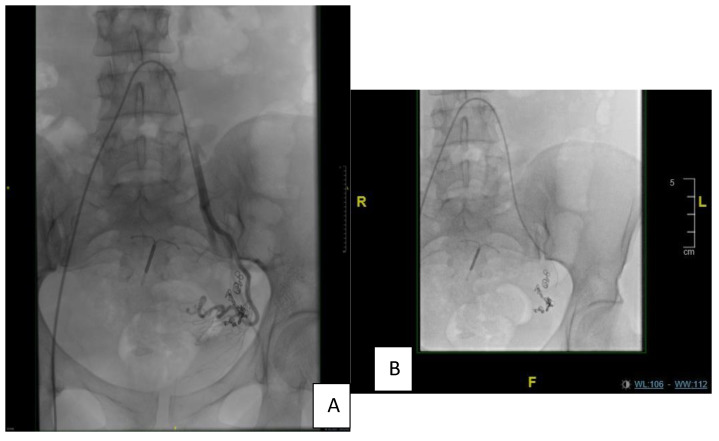
(**A**,**B**) Second Angio embolization showed total exclusion of the AVM without residual.

Second angioembolization in 2018: After informed consent was obtained from the patient, under aseptic conditions and local anesthesia, the right common femoral artery was punctured and a 6F standard sheath was inserted. Selective catheterization of the left internal iliac artery using a C2 angiocatheter followed by selective angiography, revealed recanalization of the previously embolized left superior vesical arteries approaching from the anterior division of the left internal iliac artery and supplying the known intra-resulting AVM. Selective catheterization of the left superior vesical artery using a 3F microcatheter, followed by embolization of the artery, was executed using ONYX. Final control showed total exclusion of the AVM without residual disease and with no complications (Figure 3A,B).

The patient completed almost five years of follow-up—with regular ultrasonography every six months—without recurrence, hematuria, or lower urinary tract symptoms. During this period, the patient went through a successful pregnancy for the second time. Table 2 summarizes the follow-up results.

## 3. Discussion

Mellow et al. [5] state that hemangiomas of the urinary bladder represent about 0.6% of primitive bladder tumors and can occur at any age. However, they are more frequently found in patients under 30 years old, with a slight male predominance [1]. The most common symptom is macroscopic hematuria; other symptoms include irritative voiding (LUTS) and abdominal pain [1]. Moreover, urinary bladder hemangioma is one of the benign causes of painless hematuria, which may create a challenge in diagnosis and management. Our presented case was a 38-year-old female with mild irritative LUTS and no history of macroscopic hematuria; she was referred to the urology clinic with an incidental finding using ultrasound (US) of a large bladder mass after abortion. Furthermore, this type of tumor may develop infrequently or as a part of a syndrome such as Klippel–Trenaunay–Weber syndrome or Sturge–Weber syndrome [3].

To the best of our knowledge, there have only been a few cases of pregnancy-related vertebral hemangiomas in the literature [6,7,8,9]. However, there is no case reported in the literature such as ours, which reported a case of hemangioma of the bladder with incidental findings after abortion. The physiological and hemodynamic changes, including an increase in blood volume in pregnancy, might explain the recurrence of the residual hemangioma. Additionally, the venous pressure changes from mechanical obstruction of blood flow by the gravid uterus might have had a role in the recurrence [6,8]. Nevertheless, immunohistochemical tests did not detect estrogen and progesterone receptors in the biopsy samples of the pregnancy-related hemangiomas, as reported by Schwartz et al. [10].

Cheng et al. mentioned that size ranged from 0.2–3 cm, and about 10% (two cases) were of arteriovenous hemangioma (AVM) [1]. Conversely, the diameters ranged from a few millimeters to 10 cm, with the dome being their typical location. Most bladder hemangiomas are solitary, sessile lesions that include the dome, posterior wall, and trigone [11]. A third of hemangiomas are restricted to the submucosa, while the majority penetrate the bladder wall and sporadically spread into the peri-vesical region. Their hypervascularity is visible in all imaging techniques. Hemangiomas, for instance, may exhibit enhanced activity during blood pool scintigraphy [12]. Regarding our case, there was a well-defined hypervascular soft tissue lesion measuring about 1.5 × 2.5 cm arising from the posterior wall of the urinary bladder to the left of the midline and bulging inside the urinary bladder.

The traditional methods for diagnosing bladder hemangiomas include excretory urography, cystography, and cystoscopy. Given that these lesions were vascular, a biopsy could be dangerous due to the possibility of bleeding. In addition, because they extend into the bladder submucosa, their size is sometimes underestimated. Establishing the proper diagnosis may be facilitated by using CT and the sonographic features of significant bladder wall thickening, intramural anechoic gaps, and calcification [13]. Hemangiomas exhibit a modest-to-intermediate T1 signal on magnetic resonance imaging and a distinct high T2 signal [14]. They may appear on ultrasonography as either a diffuse thickening of the bladder wall with punctate calcifications or as a confined, round, intraluminal solid mass [15]. They may also be either hyper- or hypoechoic to the bladder wall.

Regarding our case, incidentally, the ultrasound reported a large bladder mass, and the CT reported a polypoidal hypervascular lesion, as previously described, arising from the urinary bladder wall.

The diagnosis was strongly supported by cystoscopic observations of a lobulated, bluish-red, vascularized, submucosal mass in a patient with recurrent hematuria. Nevertheless, endometriosis, melanoma, and sarcoma can have comparable symptoms [16].

In our case, the diagnostic cystoscopy showed a large bluish-red, pulsatile, vascularized; submucosal mass with large, dilated, submucosal vessels; a wide-based stalk, and no active bleeding in the posterior wall of the urinary bladder, measuring about 2 × 3 cm, with negative urine cytology. Moreover, because of the probability of a difficult-to-control hemorrhage or the recurrence of bleeding after the biopsy, and considering the patient’s situation following fulguration and expected blood loss after abortion, a biopsy was not taken. Instead, the decision was taken to perform angiography and angioembolization for diagnosis and treatment.The size, location, and depth of penetration of a urinary bladder hemangioma are essential considerations for treating patients [17]. Surveillance is adequate for asymptomatic hemangiomas and minor lesions. Only when the lesions endanger organ function or the patient’s performance status—such as when hematuria causes anemia or when there is a suspicion of a malignant lesion—is therapy indicated.

Treatment varies widely; observation [2], transurethral resection, electrocoagulation, radiation, systemic steroid administration, sclerosing agent injection, interferon-α-2 therapy, YAG-laser therapy, and partial or total cystectomy are some of the alternative treatment options [11,17,18]. The most effective method for treating small cavernous hemangioma of the bladder is transurethral endoscopic surgical resection. When the lesion is small (3 cm), the risk of uncontrollable bleeding is negligible, and the results of follow-ups are positive [19,20], they can be effectively treated with a biopsy or fulguration, and neither procedure causes significant bleeding. Another efficient and minimally invasive therapeutic option is laser irradiation using neodymium: yttrium aluminum garnet (Nd: YAG), which permits complete bladder coagulation [21]. Our patient underwent her first angioembolization (2013) and was then scheduled for US every six months with regular diagnostic cystoscopy. We believe that selective angiography and angioembolization can confirm the diagnosis and appropriate treatment, especially if significant bleeding is highly suspected at the time of cystoscopy. Similarly, some reports have recommended percutaneous embolization as the first line of treatment [22,23,24].

Although urinary bladder hemangioma has a benign course, postoperative follow-up is essential for identifying tumor recurrence or residual disease; this can be achieved with flexible cystoscopy, CT scans, or even ultrasonography [20,25]. Cheng and colleagues reported that of their 19 bladder hemangioma patients, 84.2% were treated by biopsy and fulguration, and only one of their 19 patients had agreed to a partial cystectomy. No recurrent tumor was noted during a mean follow-up of 6.9 years [1]. However, after five years of follow-up, the patient in our case report developed recurrence after a successful pregnancy; an arteriovenous malformation of the left posterior wall of the urinary bladder—the same site as in the previous occasion—was detected, and angioembolization was performed for the second time by interventional radiology. Additionally, our case has been followed up for more than nine years with one recurrence, compared with the long-term follow-up mentioned by Cheng et al. and Stimac et al. (6.9 vs. 6 years), respectively.

Whereas a partial cystectomy may reduce storage function, a partial cystectomy plus bladder augmentation can preserve storage function; however, this treatment may worsen voiding function [25]. Conversely, small lesions can be treated using transurethral fulguration, angioembolization, and injection of sclerosing agents has been used with sporadic success. Nevertheless, interrupting the arterial supply will probably fail, since the new blood supply will cause hypertrophy [26]. Our reported case has almost completed another five-year period of being asymptomatic; without recurrence, less invasive management is therefore warranted. Even though hemangiomas are benign tumors, follow-up for recurrence or residual tumor is imperative [27].

## 4. Conclusions

Our reported case is a rare case of incidental bladder hemangioma after abortion. Angioembolization is a safe treatment technique, minimally invasive, and has less effect on quality of life, especially in young patients. Long-term postoperative follow-up is essential for detecting tumor recurrence or residual disease.

## Figures and Tables

**Table 1:** Summary of case management at the time of presentation in 2013.

Action	Results
**Ultrasound**	Incidental finding of large bladder mass
*Computed tomography* with contrast medium	Showed a 17.1 × 24.2 mm polypoidal hyper-vascular lesion, as previously described, arising from the urinary bladder wall (and thus unlikely to be arising from the anterior wall of the uterus).
Cystoscopy	Showed a large, bluish-red, pulsatile, vascularized submucosal mass with large dilated submucosal vessels, a wide-based stalk and no active bleeding in the posterior wall of the urinary bladder, measuring about 2 × 3 cm, with urine cytology negative.
Biopsy	Due to the vascular nature of the lesion and no active bleeding, the decision was made not to biopsy.
First angioembolization	Selective catheterization of the left internal iliac artery using a C2 angiocatheter followed by angioembolization of the left superior vesical arteries, approaching from the anterior division of the left internal iliac artery.

**Table 2:** Summary of the follow-up.

Time	Results
In 2018, recurrence after 5 years of follow-up	US showed a distended UB with a polypoidal soft tissue mass measuring 16 × 16 mm with internal vascularity, nidus in afferent and efferent vessels, and turbulence in the flow.
In 2018, second angioembolization	The patient underwent selective catheterization of the left internal iliac artery using a C2 angiocatheter; selective angiography revealed recanalization of the previously embolized left superior vesical arteries from the anterior division of the left internal iliac artery and supplying the known intra-resulting AVM. Selective catheterization of the left superior vesical artery using a 3F microcatheter, followed by embolization of the artery, was executed using ONYX.
In 2022, 5 years after the second angioembolization	Ultrasonography every six months without recurrence, hematuria, or LUTS. During this period, the patient had a successful pregnancy for the second time.

## Data Availability

The datasets used during the current study are available from the corresponding author upon request.

## References

[1] Cheng L., Nascimento A.G., Neumann R.M., Nehra A., Cheville J.C., Ramnani D.M., Leibovich B.C., Bostwick D.G. (1999). Hemangioma of the urinary bladder. Cancer.

[2] Stimac G., Dimanovski J., Katusic J., Ruzic B., Marotti M., Kraus O. (2007). A large cavernous hemangioma of the urinary bladder: Imaging of possible spontaneous regression. Eur. J. Radiol. Extra.

[3] Fuleihan F.M., Cordonnier J.J. (1969). Hemangioma of the Bladder: Report of a Case and Review of the Literature. J. Urol..

[4] Fergus K.B., Baradaran N., Tresh A., Conrad M.B., Breyer B.N. (2018). Use of angioembolization in urology: A review. Transl. Androl. Urol..

[5] Mellow M.M. (1955). Tumors of the urinary bladder: A clinicopathological analysis of over 2500 specimens and biopsies. J. Urol..

[6] Vijay K., Shetty A.P., Rajasekaran S. (2008). Symptomatic vertebral hemangioma in pregnancy treated antepartum. A case report with review of literature. Eur. Spine J..

[7] Tekkök I.H., Açìgöz B., Saglam S., Önol B. (1993). Vertebral Hemangioma Symptomatic during Pregnancy–Report of a Case and Review of the Literature. Neurosurgery.

[8] Chi J.H., Manley G.T., Chou D. (2005). Pregnancy-related vertebral hemangioma: Case report, review of the literature, and manage-ment algorithm. Neurosurg. Focus.

[9] Slimani O., Jayi S., Alaoui F.F., Bouguern H., Chaara H., Fikri G., Rachidi S.A., Houssaini N.S., Himmich M., Melhouf M.A. (2014). An aggressive vertebral hemangioma in pregnancy: A case report. J. Med. Case Rep..

[10] Schwartz T.H., Hibshoosh H., Riedel C.J. (2000). Estrogen and progesterone receptor-negative T11 vertebral hemangioma presentingas a postpartum compression fracture: Case report and management. Neurosurgery.

[11] Tavora F., Montgomery E., Epstein J.I. (2008). A Series of Vascular Tumors and Tumorlike Lesions of the Bladder. Am. J. Surg. Pathol..

[12] Ishikawa K., Saitoh M., Chida S. (2003). Detection of bladder hemangioma in a child by blood-pool scintigraphy. Pediatr. Radiol..

[13] Pakter R., Nussbaum A., Fishman E.K. (1988). Hemangioma of the Bladder: Sonographic and Computerized Tomography Findings. J. Urol..

[14] Chen M., Lipson S.A., Hricak H. (1997). MR imaging evaluation of benign mesenchymal tumors of the urinary bladder. Am. J. Roentgenol..

[15] Kogan M.G., Koenigsberg M., Laor E., Bennett B. (1996). US case of the day. Cavernous hemangioma of the bladder. Radiographics.

[16] Wong-You-Cheong J.J., Woodward P.J., Manning M.A., Sesterhenn I.A. (2006). From the Archives of the AFIP: Neoplasms of the urinary bladder: Radiologic-pathologic correlation. Radiographics.

[17] Syu S.-H., Chan K.-S., Hsiao C.-H., Chen W.-Y., Lee L.-M., Wen Y.-C. (2018). A Large Urinary Bladder Hemangioma Mimicking Urachal Cancer: A Case Report and Literature Review. Urology.

[18] Kato M., Chiba Y., Sakai K., Orikasa S. (2000). Endoscopic neodymium:yttrium aluminium garnet (Nd:YAG) laser irradiation of a bladder hemangioma associated with Klippel-Weber syndrome. Int. J. Urol..

[19] Mukai S., Tanaka H., Yamasaki K., Goto T., Onizuka C., Kamoto T., Kataoka H. (2012). Urinary bladder pyogenic granuloma: A case report. J. Med. Case Rep..

[20] Jibhkate S., Sanklecha V., Valand A. (2015). Urinary bladder hemangioma—A rare urinary bladder tumor in a child. APSP J. Case Rep..

[21] Takemoto J., Yamazaki Y., Sakai K. (2011). A case of large bladder hemangioma successfully treated with endoscopic yttrium alu-minium garnet laser irradiation. Int. J. Urol..

[22] Joyce P.F., Sundaram M., Riaz M.A., Wolverson M.K., Barner H.B., Hoffman R.J. (1980). Embolization of extensive peripheral an-giodysplasias. The alternative to radical surgery. Arch. Surg..

[23] Palmaz J.C., Newton T.H., Reuter S.R., Bookstein J.J. (1981). Particulate intraarterial embolization in pelvic arteriovenous malfor-mations. AJR Am. J. Roentgenol..

[24] Murray W.J., Fletcher M.S., Walters H.L., Packham D.A. (1986). Treatment of urethral hemangioma by selective arterial embolization. J. Urol..

[25] Lahyani M., Slaoui A., Jakhlal N., Karmouni T., Elkhader K., Koutani A., Andaloussi A.I.A. (2015). Cavernous hemangioma of the bladder: An additional case managed by partial cystectomy and augmentation cystoplasty. Pan Afr. Med J..

[26] Rosenberg J., Golimbu M., Suarez J., Morales P. (1973). Congenital Arteriovenous Malformation of the Bladder. J. Urol..

[27] Xiao L., Granberg C.F., Hull N.C. (2021). Bladder hemangioma: An arduous diagnosis of hematuria. Radiol. Case Rep..

